# Pre-viewing context and visual attention in museum scenes: an exploratory eye-tracking study

**DOI:** 10.3389/fpsyg.2026.1783603

**Published:** 2026-05-04

**Authors:** Xiaoyu Li, Jianmin Meng, Zixin Chen, Hailin Chen

**Affiliations:** 1 School of Architecture and Urban Planning, Shenzhen University, Shenzhen, China; 2 BENYUAN Design Research Center, Shenzhen, China; 3 State Key Laboratory of Subtropical Building and Urban Science, Shenzhen, China

**Keywords:** computational saliency, eye-tracking, pre-viewing context, saliency evaluation metrics, visual attention

## Abstract

Visual attention in interpretive settings is shaped not only by image saliency but also by the context viewers receive before viewing. However, how different context formats relate to attention allocation in curated visual scenes remains under-specified, and findings are often sensitive to the evaluation metric used. We conducted an exploratory free-viewing eye-tracking study in which participants viewed keyframes from museum-oriented scenes after brief pre-viewing context presented in different formats. The dataset comprises gaze recordings from 10 participants across 30 images. Attention patterns were evaluated by comparing observed gaze distributions with a saliency-model baseline and summarizing correspondence through a multi-metric profile. The results indicate systematic block-level differences in gaze-reference correspondence and variability, suggesting that pre-viewing context can be reflected in where attention concentrates, while different metrics highlight different aspects of deviation. These findings offer a compact, metric-aware way to report context-associated attention differences in small-sample studies and inform interpretive design decisions where textual and graphical cues are used to shape viewing.

## 1 Introduction

In many interpretive settings (e.g., exhibitions, heritage displays, and virtual walkthroughs), viewers often receive brief contextual information before encountering a visual scene. Such briefings may establish an interpretive frame that may be maintained in working memory and, in turn, modulate spontaneous attention allocation during viewing (Baddeley and Hitch, 1974; Miller, 1956; Baddeley, 2000, 2007). However, for computer-rendered environments it remains unclear how different context formats (text, images, or their combination) are reflected in the spatial distribution of gaze during free viewing, and how stable these observations are across evaluation metrics.

In this study we provide a preliminary characterization of how pre-viewing context is associated with attention allocation in museum-like scenes. Participants freely viewed static keyframes extracted from rendered video sequences after short briefing documents. Rather than proposing a generalisable predictive algorithm, we take gaze allocation as an indirect behavioral signature of interpretive framing and ask whether briefings are associated with consistent shifts in where viewers' attention concentrates.

To operationalize this question, we use DeepGaze III as a computational saliency baseline for static images and evaluate agreement between observed gaze distributions (sample-based gaze density maps) and saliency predictions using established saliency metrics. Following the insight that different metrics encode different assumptions and sensitivities, we report a multi-metric profile while using normalized scanpath saliency (NSS) as the primary measure and Pearson correlation coefficient (CC) as a complementary robustness check, alongside AUC, SIM, and EMD (see Section 2.2.1 for metric definitions).

### 1.1 Visual attention mechanisms

Visual attention is tightly coupled with visual perception, shaping which information is selected, prioritized, and processed in a scene (Rayner, 2009; Wolfe and Horowitz, 2017; Lupyan et al., 2020). A common distinction is between top-down and bottom-up influences. Top-down attention reflects selection that is dependent on goals and prior knowledge, shaped by expectations, and supported by short-term maintenance processes such as working memory (Bleckley et al., 2003; Cabeza et al., 2004; Firestone and Scholl, 2015, 2016; Lupyan et al., 2020). Bottom-up attention, in contrast, is driven by stimulus properties – such as contrast, color, brightness, and motion-that can attract attention automatically (Adelson, 1993; Corbetta and Shulman, 2002; Wolfe and Horowitz, 2004; Hollingworth and Hwang, 2013; Firestone and Scholl, 2015). Together, these influences allow attention to remain both goal-directed and responsive to salient features in the environment.

The study of attention has developed through several theoretical paradigms. Posner's spotlight model frames attention as a selective enhancement mechanism that facilitates processing at attended locations (Posner, 1980; Posner and Petersen, 1990). Feature-integration theory further suggests that basic attributes can be processed in parallel, whereas attention is required to bind features into coherent perceptual units (Treisman and Gelade, 1980; Treisman, 1998). These accounts motivate computational approaches that model which regions are likely to be attended under stimulus-driven conditions.

Building on these works, the Itti-Koch saliency framework introduced a computational account of bottom-up salience, predicting conspicuous regions from feature contrasts in basic image properties (Koch and Ullman, 1987; Itti et al., 1998; Itti and Koch, 2000, 2001). However, classical saliency models are limited in their ability to incorporate higher-level influences such as task relevance and semantic meaning, which reduces their explanatory power in complex real world scenes (Tatler et al., 2011). Recent deep learning models, including DeepGaze III (Kümmerer et al., 2022, 2014, 2016), partially address this limitation by incorporating semantic information about objects and scenes to improve prediction of human fixations.

### 1.2 Pre-viewing textual and pictorial context in attention allocation

Saliency models provide a useful baseline for predicting stimulus-driven attention, but viewing in interpretive settings is also shaped by information provided before a scene is encountered. Brief pre-viewing materials can set an interpretive frame and influence subsequent free viewing (Bae and Luck, 2017). This influence is often framed as top-down selection supported by short-term representations that can guide attention, such as working memory contents acting as attentional templates (Soto et al., 2005; Olivers, 2008; Chun, 2011; Soto et al., 2006; Olivers et al., 2011; Woodman et al., 2013; Olivers and Roelfsema, 2020). In the present study, working memory is not independently measured or manipulated; it is therefore referenced only as an interpretive construct rather than a tested mechanism. Accordingly, we review how text and images presented before viewing can affect attention allocation in later scene viewing.

Textual information can guide attention by shaping semantic expectations and narrowing what viewers treat as relevant (Lupyan et al., 2020). Compared with purely stimulus-driven orienting, textual cues often engage interpretation and goal-directed selection, influencing where viewers choose to look based on comprehension and meaning (Lewis et al., 2019; Lupyan and Lewis, 2019). When the text is concise and aligned with the scene (e.g., the briefing highlights an object, motif, or spatial cue that is visibly present in the upcoming frames, allowing viewers to prioritize corresponding regions during viewing), it can help viewers sample information more selectively; when it is ambiguous, or poorly structured, it may compete with visual exploration and increase processing demands, producing more dispersed viewing patterns (Lavie and Tsal, 1994). Related evidence from applied visual search in visually complex displays shows that both target salience and clutter can shape search efficiency and viewing behavior, even when the target representation is strong (Hicks and Still, 2019). Moreover, salience can continue to guide attention under heavy top-down guidance in complex displays (Still and Still, 2019). Beyond search tasks, early visual attention has been shown to bias subsequent preference and choice in value-based decisions, underscoring the diagnostic value of time-resolved viewing (Milosavljevic et al., 2012; Gidlöf et al., 2017).

Pictorial information, by contrast, provides concrete visual signals that can prime expectations about objects, spatial layout, and relevant regions (Kristjánsson and Driver, 2008; Gurtner et al., 2021). These image-based cues can also attract gaze through basic visual properties such as contrast and color, but this initial attraction does not necessarily translate into sustained interpretive focus (Adelson, 1993; Hollingworth and Hwang, 2013) for instance, sustained viewing of meaning-relevant elements such as the focal artifact, its symbolic motifs, or accompanying interpretive cues, rather than only transient orienting to high-contrast hotspots. In complex scenes, visually rich content may broaden exploration and redistribute attention across a larger set of regions (Wolfe and Horowitz, 2004; Rosenholtz et al., 2007).

Text and images can also interact in ways that are complementary or competing. When the two are coherent, text can anchor interpretation while images provide concrete visual examples, potentially supporting more stable attention allocation (Mayer and Moreno, 2003). When they are misaligned or overly dense, viewers may need to allocate attention across competing cues, which can increase variability in where attention concentrates (Chandler and Sweller, 1991; Sweller, 1994; de Jong, 2010). Building on this perspective, the present study compares three pre-viewing context formats (text only, images only, and text and images combined) and examines whether they are associated with consistent differences in gaze distribution relative to a computational saliency baseline, assessed across multiple evaluation metrics.

### 1.3 Metrics for evaluating gaze saliency correspondence

To describe how attention allocation differs across pre-viewing context formats, we quantify the correspondence between empirical sample-based gaze density maps and saliency predictions from a computational baseline. We report a complementary metric profile comprising NSS as primary agreement measure and CC as robustness check, while area under the ROC curve (AUC), similarity (SIM), and earth mover's distance (EMD) are retained as secondary reference measures to characterize how departures from the baseline are expressed across classification, shared spatial layout, and map-to-map distance.

Moreover, considering pre-viewing context may not only shift where gaze concentrates but also how broadly viewing is distributed over time, we then report Gaze Entropy as an empirical dispersion descriptor computed from the empirical gaze density map (Ahn et al., 2017). Those metric definitions and implementation details are further provided in Section 2.2.1. As these measures rely on different assumptions and can respond differently to smoothing, center bias, and distributional spread, we interpret convergence across metrics rather than relying on any single score.

### 1.4 Saliency predictions as a computational baseline

To compute the evaluation metrics introduced above, we require a saliency prediction generated by a computational model as a reference for comparison with empirical gaze distributions. While cognitive theories and eye-tracking studies offer explanatory accounts of attention, computational saliency models provide an explicit and scalable way to generate baseline predictions of likely gaze density on static images (Itti et al., 1998; Itti and Koch, 2001; Judd et al., 2012; Borji and Itti, 2013; Riche et al., 2013; Rahman et al., 2021; Khan et al., 2019). In visually complex applied displays, computational salience maps have been used as an objective way to characterize salience gradients and to explain why salient regions can continue to guide attention even under goal-driven viewing (Still and Still, 2019; Hicks and Still, 2019; Still, 2025).

Saliency models produce saliency maps that assign higher values to regions expected to attract attention under stimulus-driven viewing. Early approaches estimated saliency from contrasts in basic image features (e.g., intensity, color, and orientation), providing a principled baseline for where attention might concentrate in the absence of richer contextual influences (Itti et al., 1998; Itti and Koch, 2000, 2001; Peters et al., 2005; Le Meur et al., 2006; Bernhard et al., 2007; Bruce and Tsotsos, 2009; Lin et al., 2013; Duan and Wang, 2015). More recent data-driven models incorporate higher-level information (e.g., objects and scene structure) and have improved fixation prediction in complex scenes (Judd et al., 2012; Kümmerer et al., 2022).

In this study, we use DeepGaze III to generate saliency predictions for each static image and treat these predictions as a computational baseline rather than a definitive reference for attention. By comparing DeepGaze III outputs with observed gaze distributions under different briefing scenarios, we characterize how gaze-baseline correspondence changes, and report this change descriptively across multiple evaluation metrics.

### 1.5 Research gap and aims

Despite substantial progress in saliency modeling and gaze prediction, evaluation practice still faces two practical limitations when studies move from natural images to authored, rendered interpretive scenes. First, prior work often reports a single metric or combines multiple metrics without clarifying what each score is sensitive to, even though different metrics can respond differently to map properties such as variance, location bias, and the balance between false positives and false negatives. This makes it difficult to interpret agreement between model predictions and human gaze allocation as a stable statement rather than one that depends on the chosen metric. Second, in interpretive contexts (e.g., exhibitions and heritage media), viewers commonly receive brief contextual information before viewing, yet it remains unclear how different pre-viewing context formats are reflected in the spatial distribution of gaze in rendered scenes, and how stable these observations are across evaluation metrics.

To address these gaps, this study uses free-viewing eye tracking on static frames from rendered museum-context scenes and treats DeepGaze III predictions as a computational baseline. We report a transparent multi-metric evaluation profile, with NSS as the primary measure, to characterize how gaze allocation and agreement with the baseline change across pre-viewing context blocks. This leads to the central research question:

- How can a multi-metric evaluation profile be used to describe changes in gaze allocation and agreement with a saliency baseline across different pre-viewing context formats in rendered interpretive scenes?

The goal is an exploratory and design-oriented characterization rather than a generalisable predictive model, providing an interpretable reporting template for similar studies with a limited sample size.

## 2 Methods

### 2.1 Experimental design and evaluation framework

To examine whether pre-viewing context is associated with changes in attention allocation, we used a within-participant block design comprising three briefing scenarios (A-C). Each scenario consisted of a short briefing document followed by free viewing of 10 static keyframes extracted from rendered sequences. The three scenarios differed in the modality of briefing information (text only, images only, and text and images combined) and thematic object category (pottery, mask, bouquet). Hence, cross-scenario comparisons are treated as exploratory and are discussed with this confound acknowledged.

Ten participants (*N* = 10) were recruited for this exploratory study from Shenzhen University (undergraduate and postgraduate students, and university staff and faculty). Background information of participant relevant to scene viewing is reported in Table 1, including age, role, visual expertise, annual museum visiting frequency, prior VR experience, and self-reported familiarity with the three object categories used in the briefing blocks.

**Table 1:** Background information of participant relevant to scene viewing.

ID	Age	Role	Visual expertise	Museum visits per year	VR experience	Object familiarity (pottery/mask/bouquet)
1	22	UG	None	1	None	Low/Low/Low
2	24	PG	None	2	Limited	Low/Low/Low
3	29	Staff	Limited	1	None	Low/Moderate/Low
4	21	UG	None	0	None	Low/Low/Low
5	27	PG	None	3	Limited	Moderate/Low/Low
6	32	Staff	None	1	None	Low/Low/Moderate
7	23	UG	None	2	Limited	Low/Low/Low
8	35	Staff	Limited	1	None	Low/Moderate/Low
9	26	PG	None	0	None	Low/Low/Low
10	42	Staff	None	2	Limited	Low/Low/Moderate

“*UG” means undergraduate. “PG” means postgraduate. “Museum visits per year” means self-reported annual frequency of visiting museums or galleries*.

**Figure 1:**
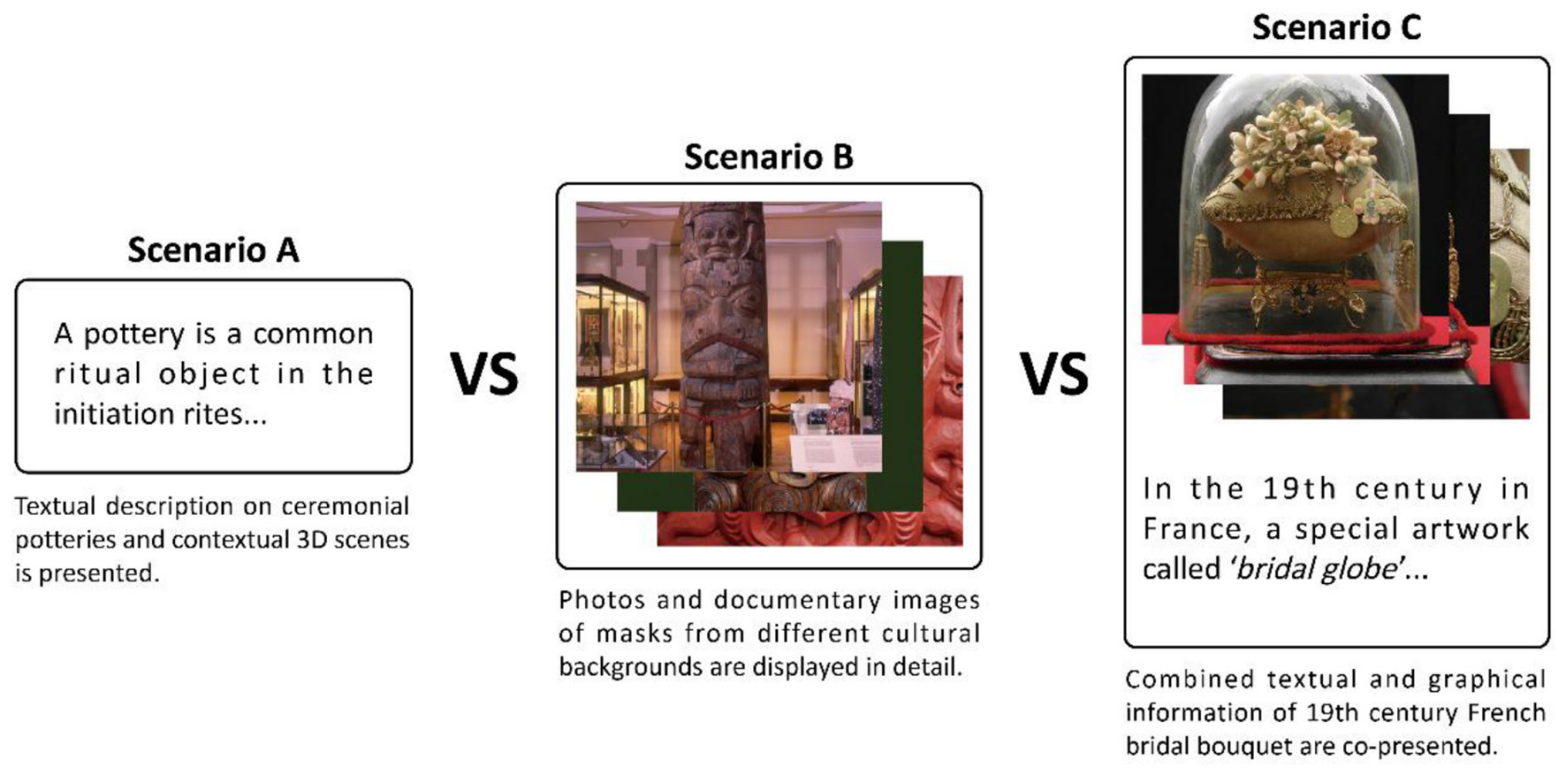
Three pre-viewing context conditions (Scenarios A-C) and the block structure of the experiment. Scenario B adopted from: Cambridge Museum of Archaeology and Anthropology, ‘Totem pole', Creative Commons license (CC BY-NC-ND 4.0), https://collections.maa.cam.ac.uk/objects/487619/; Skecthfab, ‘Maori', CC BY 4.0, https://sketchfab.com/3d-models/maori-78535f42f67c4314a3b4ac864f2a22bd; Cenk Unver, Dreamstime, ‘Maori Mask Detail', Standard Dreamstime Royalty-Free License (downloaded via subscription plan), https://www.dreamstime.com/royalty-free-stock-photos-maori-mask-image19109058. Scenario C adopted from: Alienor.org, ‘Bouquet de mariage', CC Attribution-NonCommercial-NoDerivs, https://sketchfab.com/3d-models/bouquet-de-mariage-92a843612e1e42159aebba1c617c0622.

This experiment formed part of a broader research project entitled ‘Interference of Text and Image Information on Visual Attention', under which the ethical approval was obtained. The study was approved by the Medical Ethics Committee of the Medical School, Shenzhen University (VT Project No. PN-202500038), and all participants provided written informed consent prior to participation. Given the sample size, analyses emphasize descriptive summaries, with non-parametric tests used where appropriate. Each participant completed three sequential briefing scenarios (Scenarios A-C, as shown in Figure 1). Because briefing format varies along with object category in the current design and blocks were not counterbalanced, comparisons across scenarios are interpreted as descriptive block-level patterns rather than independent causal effects of modality.

During the experiment, participants completed three blocks in a fixed order (A-C). At the beginning of each block, they reviewed a briefing document designed to provide contextual framing for the upcoming images:

- Scenario A (pottery-text only): a text briefing describing ritual and symbolic meanings of pottery and breakage as a social ritual metaphor.
- Scenario B (mask- image only): an image only briefing consisting of concrete object photographs (no text).
- Scenario C (bouquet-text and image combined): a combined briefing providing both abstract meaning (text) and concrete visual references (images) about a nineteenth-century French bridal display object.

After the briefing, participants freely viewed 10 static images (keyframes) from the corresponding rendered scenes. Each image was displayed for 5-s and advanced automatically. Eye movements were recorded at 120 Hz using a VR HMD eye tracker (HTC VIVE Pro). Participants were instructed to view each image naturally, without an explicit search or memory task, so that spontaneous points of interest could be observed under contextual framing.

Each 5-s trial produced up to 600 gaze samples at 120 Hz. After removing invalid samples (e.g., tracking loss), we constructed sample-based gaze density maps in image coordinates by accumulating the remaining gaze samples into a 2D histogram and applying isotropic Gaussian kernel smoothing (σ = 15 pixels) to obtain a continuous density surface. All map-construction steps (binning, smoothing, and normalization) were applied identically across scenarios and time windows. For time-binned analyses, the same procedure was applied separately to samples falling within the early (0–1 s) and late (1–5 s) windows. As a robustness check against map-construction choices in VR, we repeated the core analyses using (i) all valid gaze samples and (ii) a conservative subset of gaze samples labeled as fixations (as classified by the HTC VIVE pro eye-tracker software).

**Figure 2:**
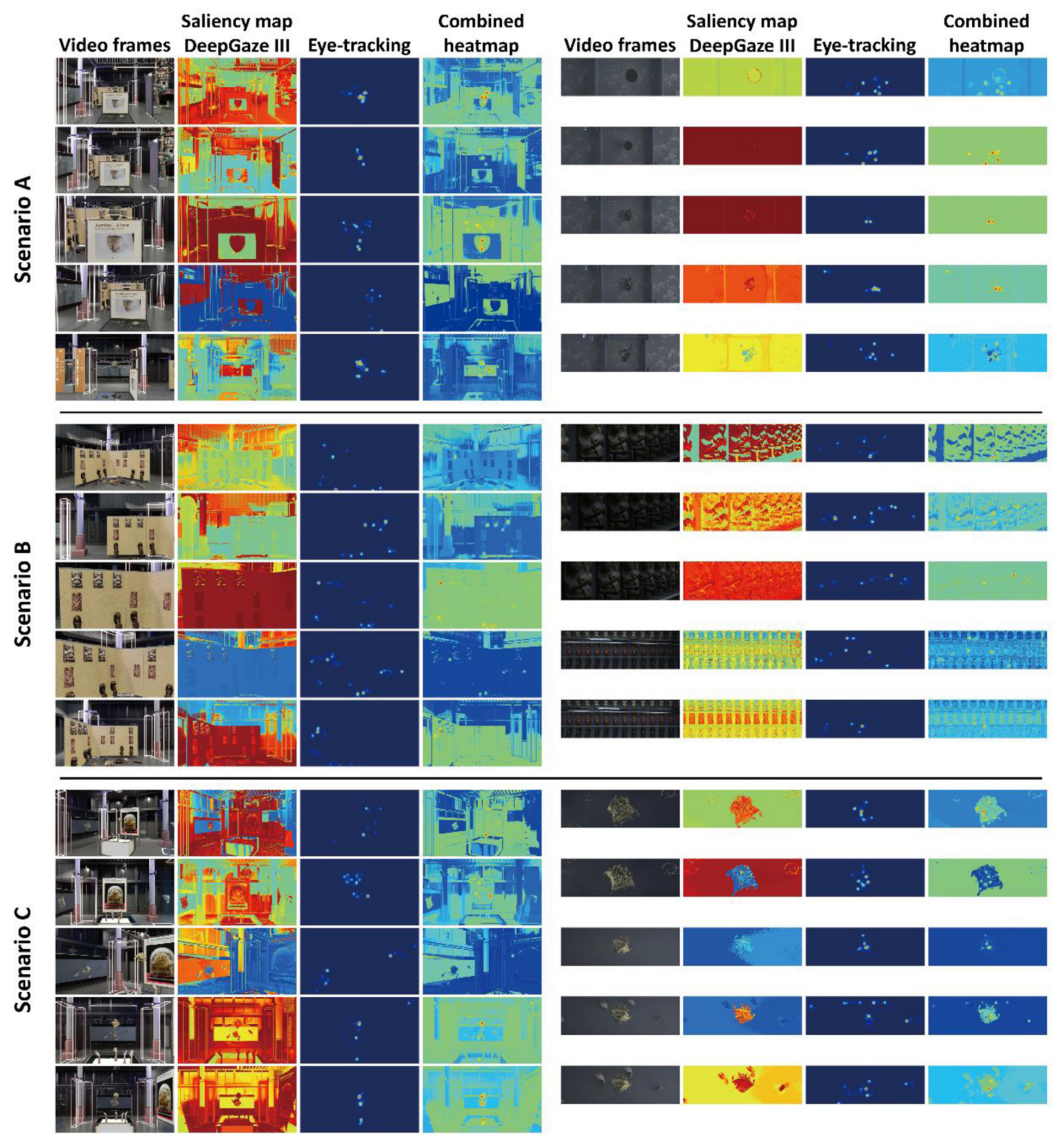
Overview of the rendered stimuli and corresponding gaze-saliency correspondence visualizations – rendered keyframes, DeepGaze III predictions, empirical gaze density maps, and overlay images.

Stimuli were generated by reconstructing 3D objects and scenes using Rhinoceros and Cinema4D, rendering first-person walkthrough sequences and stylised CG sequences, and extracting two sets of five consecutive keyframes per block (10 images per block), which were presented as static 2D images in the HMD. We used the publicly available DeepGaze III model as a computational saliency baseline for these static images (Figure 2). The model was not retrained on the present stimuli. Therefore, results are interpreted as changes in gaze-baseline correspondence across briefing blocks, rather than as an absolute test of model validity for this specific domain.

### 2.2 Metric-based evaluation framework for gaze-saliency deviation

#### 2.2.1 Selection and Definition of evaluation metrics

To quantify how empirical gaze patterns relate to a computational saliency baseline under each briefing scenario, we computed five widely used saliency evaluation metrics–CC, NSS, AUC, SIM, and EMD (Duncan and Sarkar, 2012; Riche et al., 2013; Wang and Shen, 2017; Bora et al., 2020; Hosseini et al., 2024). These metrics capture complementary aspects of gaze-saliency correspondence, including map-level similarity (CC, SIM), gaze-location-centered agreement (NSS), discriminability between gaze and non-gaze locations (AUC), and distributional distance between maps (EMD).

In addition, to characterize how spatially concentrated or dispersed empirical viewing was within a time window, we report an empirical dispersion descriptor computed from the empirical gaze map (Gaze Entropy). Gaze Entropy is interpreted descriptively as reflecting the spread and complexity of the empirical gaze distribution, and it is reported alongside baseline-fit metrics rather than being treated as a saliency-model evaluation metric.

CC quantifies the linear correspondence between predicted and empirical saliency maps, whereas NSS summarizes normalized saliency values at observed gaze locations, providing a gaze-location-centered measure that is comparable across images (Peters et al., 2005; Riche et al., 2013). AUC assesses discriminability between gaze and non-gaze locations (Riche et al., 2013; Wang and Shen, 2017). SIM measures overlap between normalized saliency distributions, and EMD characterizes distributional distance between maps (Rubner et al., 1998, 2000; Wang and Shen, 2017). Entropy is included as a compact descriptor of saliency-map dispersion (Duncan and Sarkar, 2012).

#### 2.2.2 A descriptive composite deviation score

To provide a compact summary for descriptive reporting, we computed a composite deviation score (CDS). For each participant, each baseline-fit metric (CC, NSS, AUC, SIM, EMD) was min-max normalized to the range [0, 1] across all 30 images. For agreement-type metrics (CC, NSS, AUC, SIM), we converted normalized agreement to deviation as 1 − *m*′, so that larger values consistently indicate larger deviation from the saliency baseline. For EMD, the normalized value *m*′ was used directly because larger EMD already indicates greater distributional distance. CDS is not intended as an optimal weighting scheme or a psychological scale. Instead, it is reported only as a compact descriptor alongside the full metric profile and is interpreted descriptively within participants across scenarios.

#### 2.2.3 Composite deviation score (CDS)

For image *i*, the composite deviation score is defined as:

$CDS_{i}=\frac{\left(1-{CC^{\prime }}_{i}\right)+\left(1-{NSS^{\prime }}_{i}\right)+\left(1-{AUC^{\prime }}_{i}\right)+\left(1-{SIM^{\prime }}_{i}\right)+{EMD^{\prime }}_{i}}{5}$

where the prime (′) denotes min-max normalization of each metric to [0, 1] within each participant across all 30 images. Higher CDS indicates larger overall deviation between empirical gaze and the computational saliency baseline under a given briefing scenario, in the sense of reduced agreement on agreement-type measures and increased distributional distance.

### 2.3 Time-binned and direct empirical analyses

Because context effects are plausibly strongest early in viewing, we conducted time-binned analyses of gaze maps. For each 5-s trial, gaze samples were split into an early window (0–1 s) and a late window (1–5 s), and empirical gaze density maps were constructed within each window using the same preprocessing and normalization as in the full-trial maps.

To directly test whether empirical gaze allocation differs across briefing scenarios (independent of any computational baseline), we performed direct empirical contrasts. For each participant and each time window, we aggregated gaze samples across the 10 images within each scenario to form a block-level empirical gaze map for Scenario A, Scenario B, and Scenario C. We then computed pairwise map similarity/distance between scenarios (A-B, A-C, B-C) using the same map-level measures (CC, SIM, EMD). These direct empirical contrasts quantify scenario differences in observed gaze distributions without reference to DeepGaze III.

For statistical reporting, within-participant comparisons across scenarios used a Friedman test. Where relevant, follow-up pairwise comparisons used paired Wilcoxon signed rank tests with Holm-Bonferroni adjustment. Given the exploratory design that briefing format co-varies with object category and blocks were not counterbalanced, results are interpreted as descriptive block-level differences rather than isolated causal effects of modality.

## 3 Results

### 3.1 Result overview

**Figure 3:**
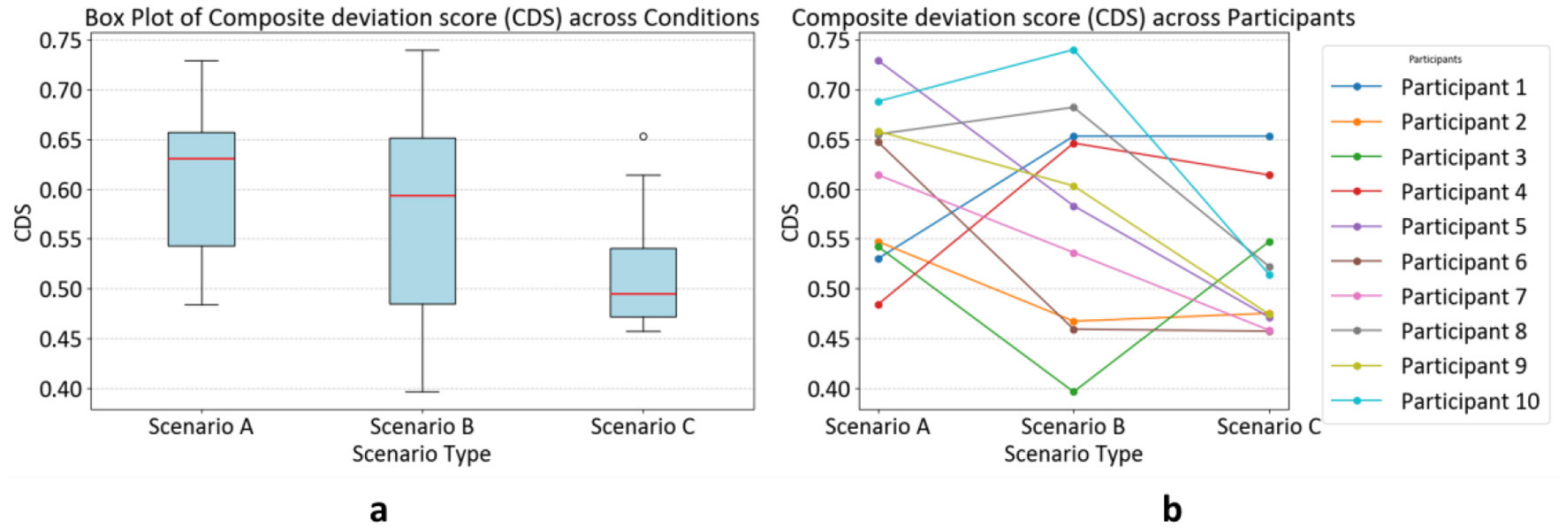
Participant-level CDS across scenarios (A-C). **(a):** Distribution of CDS values. **(b)**: Participant trajectories across scenarios (each line represents one participant).

Table 2 summarizes participant-level scenario mean CDS values (higher values indicates larger deviation from the saliency baseline, as mentioned in Section 2.2.3). Using the five-metric CDS computed over the full 5-second viewing window, Scenario A shows the largest overall deviation (median = 0.614; mean = 0.609), Scenario B is intermediate (median = 0.583; mean = 0.576), and Scenario C shows the smallest deviation (median = 0.514; mean = 0.519). These participant-level values are visualized, and substantial individual differences are highlighted in Figure 3. Across participants, CDS is lower in Scenario B than in Scenario A for six of 10 participants, and lower in Scenario C than in Scenario B for seven of 10 participants. Four of 10 participants show a monotonic A > B > C trajectory. Given that briefing format co-varies with object category and block order in the present design, these results are reported as descriptive block-level differences in gaze-baseline deviation across blocks rather than as evidence for causal effects of briefing modality.

**Table 2:** Composite deviation score (CDS) of ten participants across three briefing scenarios (A-C). Higher values indicate larger deviation from the DeepGaze III saliency baseline.

Scenarios	Participants									
	1	2	3	4	5	6	7	8	9	10
Scenario A	0.530	0.547	0.542	0.484	0.729	0.647	0.614	0.655	0.658	0.688
Scenario B	0.653	0.467	0.396	0.646	0.583	0.459	0.536	0.682	0.603	0.740
Scenario C	0.653	0.475	0.547	0.614	0.471	0.457	0.458	0.522	0.474	0.514

### 3.2 CDS distribution across briefing blocks

Figure 3 visualizes participant-level CDS values across the three briefing scenarios. Differences are interpreted descriptively as changes in deviation from the saliency baseline associated with the pre-viewing context. Given the design constraints aforementioned (scenario content and block order are not independent), we do not attribute cross-scenario differences to briefing modality alone. Instead, we treat CDS patterns as compact descriptive summaries that can inform future counterbalanced designs and additional behavioral measures. To directly test whether gaze allocation differs across scenarios beyond baseline-fit differences, we next report direct empirical gaze-map contrasts (gaze vs. gaze), including time-binned (early vs. late) analyses.

### 3.3 Direct empirical gaze-map contrasts across scenarios

**Figure 4:**
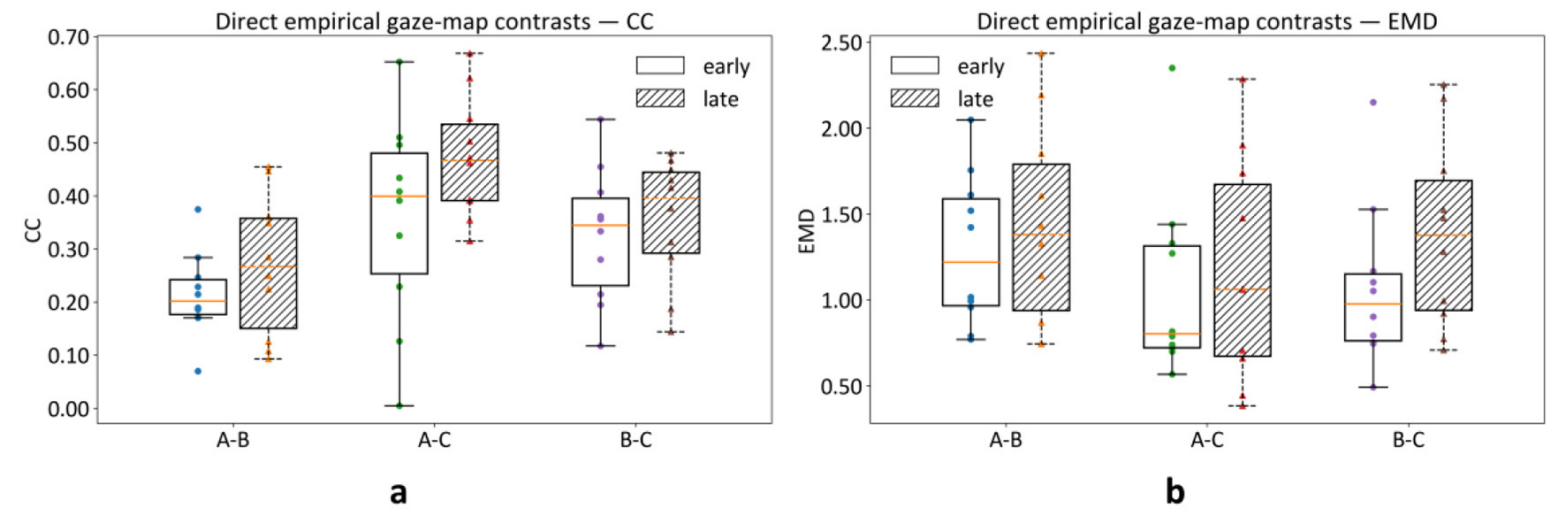
Direct empirical gaze-map contrasts across scenarios. Pairwise differences between block-level empirical gaze maps (A–B, A–C, B–C) are shown for Early (0 – 1 s) and Late (1 – 5 s) windows. **(a):** Map similarity (CC). **(b):** Distributional distance (EMD). Points represent participants and boxplots summarize the across-participant distribution.

To test whether empirical gaze allocation differs across scenarios independently of any computational baseline, we performed direct empirical comparisons between block-level gaze maps (Scenario A vs. B, A vs. C, and B vs. C). For each participant and each time window, gaze samples were aggregated across the 10 images within each scenario to form three scenario-level empirical gaze maps; pairwise gaze-map similarity and distance (A-B, A-C, B-C) were then computed using CC and EMD (Figure 4).

Direct empirical contrasts using map-level CC revealed reliable differences across scenario pairs in both the early window (Friedman χ^2^(2) = 7.80, *p* = 0.02, Kendall's W = 0.39) and the late window (χ^2^(2) = 11.40, *p* = 0.0033, W = 0.57; Table 3). Follow-up pairwise comparisons (paired Wilcoxon tests with Holm correction) indicated that the A-B contrast differed from A-C and from B-C in both time windows (all *p*_Holm ≤ 0.041). This pattern is directly visible in Figure 4a, where A-B shows the lowest CC (least similar gaze distributions) in both early and late windows. Figure 4b provides convergent evidence from a distance-based measure (EMD), illustrating that scenario differences are also expressed as distributional distance between empirical gaze maps. Together, these results demonstrate that scenario differences are observable at the level of empirical gaze distributions, rather than being reducible to baseline-fit differences alone.

**Table 3:** Non-parametric statistical summary for (i) direct empirical gaze-map contrasts across scenarios (A–C), (ii) time-binned gaze-DeepGaze correspondence (NSS and CC), and (iii) empirical dispersion of gaze maps (Gaze Entropy). Friedman tests were used for within-participant comparisons across scenarios within each time window (Early: 0 – 1 s; Late: 1 – 5 s). Follow-up pairwise comparisons used paired Wilcoxon signed-rank tests with Holm correction. For direct empirical CC, the A–B contrast differed from A–C and from B–C in both early and late windows (all *p*_Holm ≤ 0.041). For late Gaze Entropy, Scenario B exceeded Scenario A and Scenario C (*p*_Holm ≤ 0.0078).

Analysis	Metric	Time window	Test	χ^2^(df)	Kendall's W	*p*
Direct empirical contrasts	CC	Early (0-1 s)	Friedman test	7.8	0.39	0.020
Direct empirical contrasts	CC	Late (1-5 s)		11.4	0.57	0.003
Gaze-DeepGaze correspondence	CC	Early (0-1 s)		3.8	0.19	0.150
Gaze-DeepGaze correspondence	CC	Late (1-5 s)		0.2	0.01	0.905
Gaze-DeepGaze correspondence	NSS	Early (0-1 s)		3.8	0.19	0.150
Gaze-DeepGaze correspondence	NSS	Late (1-5 s)		0.8	0.04	0.670
Empirical dispersion	Gaze Entropy	Early (0-1 s)		4.2	0.21	0.122
Empirical dispersion	Gaze Entropy	Late (1-5 s)		12.6	0.63	0.002

*These comparisons reflect bundled block-level differences (briefing format co-varies with object category and fixed block order) rather than isolated causal effects of modality*.

### 3.4 Time-binned gaze-DeepGaze correspondence and empirical dispersion

**Figure 5:**
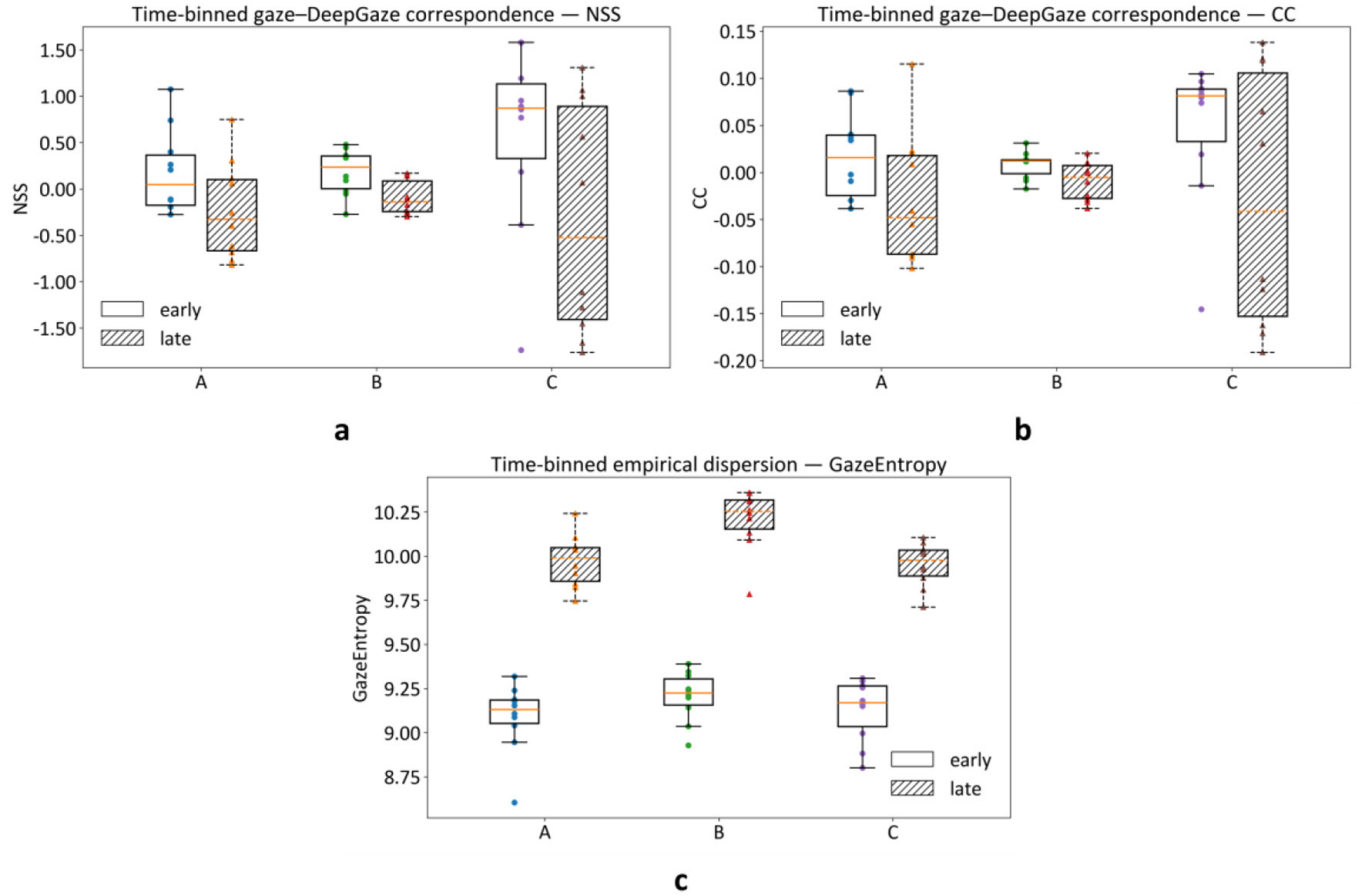
Time-binned gaze-baseline correspondence and empirical dispersion. Participant-level gaze-DeepGaze correspondence is shown for **(a)** NSS and **(b)** CC in Early (0 – 1 s) and Late (1 – 5 s) windows**. (c)** Empirical dispersion of gaze maps (Gaze Entropy) shows how broadly gaze is distributed within each time window. Points represent participants and boxplots summarize across-participant distributions.

We next examined how scenario differences manifest relative to the DeepGaze III saliency baseline over time. Figure 5a-b report time-binned gaze-DeepGaze correspondence for the primary agreement measures (NSS and CC) in early (0 – 1 s) and late (1 – 5 s) viewing windows, aggregated at the participant level (participants as the statistical unit). Omnibus tests did not indicate strong scenario separation on NSS or CC within either time window in this sample (Table 3). Nevertheless, the time-binned profiles are diagnostically useful. As is shown in Figures 5a, b, Scenario C shows higher correspondence early but a marked reduction in correspondence later, whereas Scenario B tends to remain closer to zero in the late window. These descriptive profiles complement the direct empirical contrasts reported above by indicating when deviations from baseline are most apparent.

In addition to baseline-fit measures, we assessed the dispersion of empirical gaze distributions using Gaze Entropy computed from the empirical gaze maps (Figure 5c). Gaze Entropy showed a clear scenario effect in the late window (Friedman χ^2^(2) = 12.60, *p* = 0.00184, W = 0.63; Table 3), with follow-up comparisons indicating higher dispersion in Scenario B than in Scenarios A and C (*p*_Holm ≤ 0.0078). This pattern suggests that scenario differences can involve not only shifts in where attention concentrates, but also changes in how broadly gaze is distributed over time.

### 3.5 Metric sensitivity and robustness across measures

**Figure 6:**
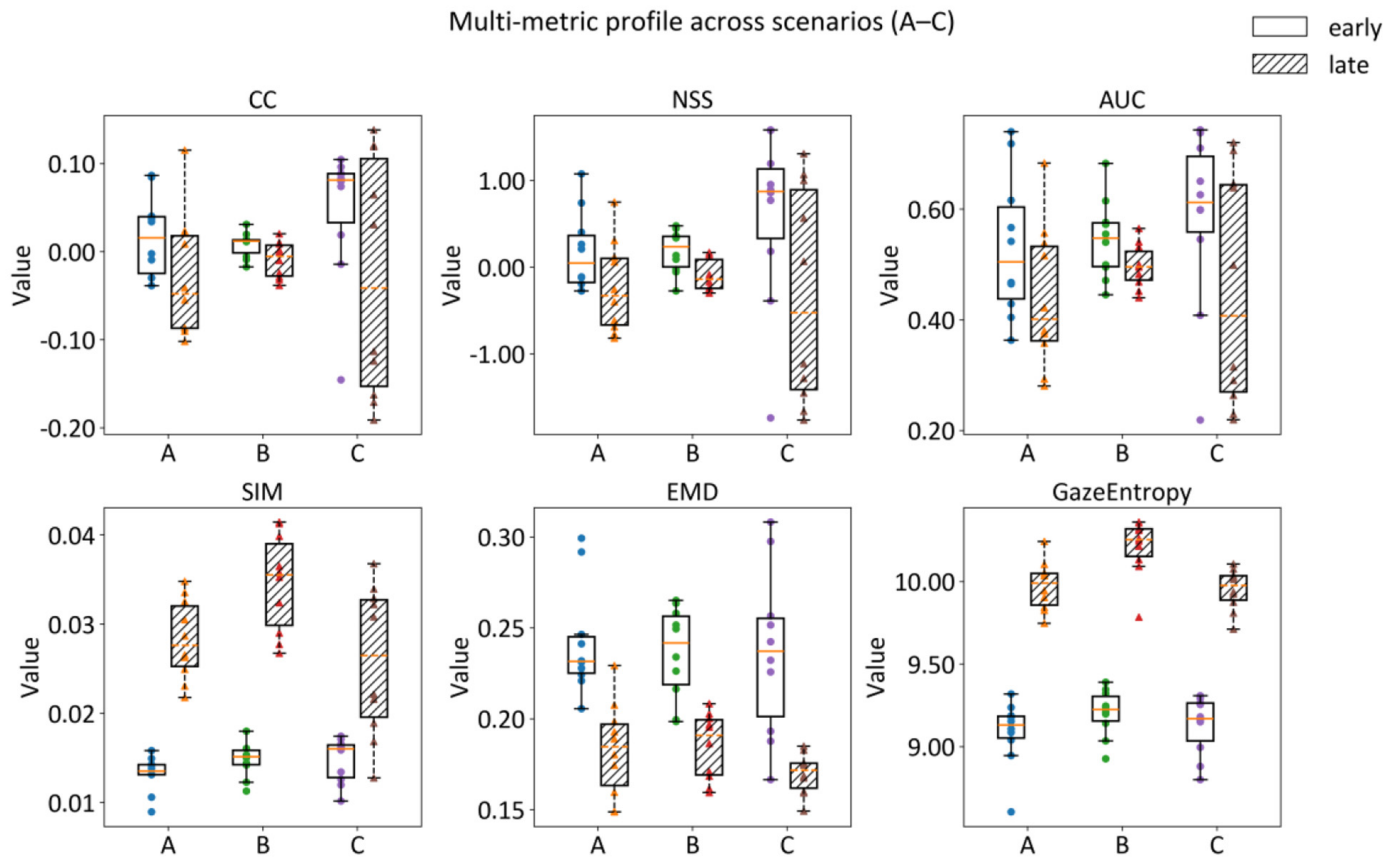
Multi-metric profile across scenarios (A-C) and time windows. Distributions of CC, NSS, AUC, SIM, EMD, and empirical dispersion (Gaze Entropy) are shown at the participant level (each point represents one participant's mean across the 10 images in that scenario), separately for early (0 – 1 s) and Late (1 – 5 s) windows.

Because saliency metrics differ in assumptions and error sensitivities (Bylinskii et al., 2019), we report a metric profile rather than relying on a single score. NSS is treated as the primary agreement measure and CC as a complementary robustness check. The remaining metrics (AUC, SIM, EMD) provide interpretive context for whether scenario differences reflect shifts in discriminability, distributional overlap, or spatial distance between maps. Although Figures 4–5 focus on the measures most directly tied to the present study question, Figure 6 retains AUC and SIM because they remain useful for nearby exhibition-viewing questions beyond the present emphasis on NSS and CC. In particular, AUC is informative for studies asking whether preview cues or interpretive aids draw gaze more selectively to intended regions, whereas SIM is informative for studies asking whether different briefing schemes preserve or re-organize the broader spatial layout of attention across a exhibitory display. Reporting them therefore keeps the metric profile relevant not only to the present analysis, but also to closely related museum and interpretive-viewing studies with a stronger focus on cueing or scene-level layout effects.

Across analyses, the key contribution is not a single-metric ordering but the convergence between (i) direct empirical gaze-map contrasts demonstrating scenario-dependent differences in observed gaze distributions (Section 3.3) and (ii) time-binned profiles describing when and how these differences relate to the DeepGaze baseline and to empirical dispersion (Section 3.4). Together, this multi-metric reporting strategy helps distinguish changes that are reflected across measures from changes that primarily reflect dispersion or distributional spread.

## 4 Discussion

### 4.1 Theoretical contribution

This study contributes an initial descriptive account of how pre-viewing contextual materials are associated with attention allocation in rendered interpretive scenes. By combining free-viewing eye tracking with a computational saliency baseline and a multi-metric reporting strategy, we demonstrate a practical way to describe how gaze patterns depart from a baseline prediction under different pre-viewing contexts. These findings are discussed as descriptive block-level differences in gaze-baseline correspondence.

Across the three blocks, the results suggest two related but distinguishable patterns in subsequent viewing. One concerns where gaze is allocated, as captured by empirical gaze distributions. The other concerns how broadly gaze is distributed over time, as captured by dispersion. Section 3.3 shows that scenario differences are visible at the level of empirical gaze distributions without relying on the baseline model. In both early and late windows, the Scenario A (text-only pre-viewing) and Scenario B (image-only pre-viewing) contrast exhibits the clearest separation in empirical allocation patterns at the participant level. Section 3.4 adds a temporal perspective by showing that the diagnostic value of correspondence is stronger early, whereas later viewing is characterized more by differences in dispersion, with the image only block showing the strongest late window spread. Taken together with the full window CDS summary in Sections 3.1 and 3.2, the overall picture is that contextual effects are better treated as time sensitive patterns that can involve both allocation shifts and changes in dispersion.

As mentioned in Section 1.2, stimulus-driven salience can remain robust under strong top-down guidance, and the present block-level differences are also compatible with scene-viewing accounts in which global context and meaning guide attention beyond low-level salience. Contextual guidance models emphasize that global scene information constrains selection during viewing and search, and meaning-map work shows that semantic informativeness can predict where people look even when salience is controlled (Torralba et al., 2006; Henderson and Hayes, 2017, 2018; Pedziwiatr et al., 2021). Relatedly, classic findings show that top-down demands can rapidly override stimulus-driven saliency and that semantically inconsistent objects can attract early looks, both of which can produce systematic departures from saliency-model predictions (Einhäuser et al., 2008; Loftus and Mackworth, 1978).

### 4.2 Practical implications

From an interpretive design perspective, the results suggest that pre-viewing materials can act as an attention priming layer that shapes how viewers enter a scene. Considering the present paradigm isolates a short 5 s viewing episode, it is especially relevant to curatorial situations in which visitors first encounter a scene after a preview panel, introductory label, image cue, or digital preface. At that scale, the method can help designers test whether a preview supports immediate orienting toward intended elements, preserves a more stable initial reading, or instead disperses attention across the scene. The most consistent differences appear early, while later viewing can show changes in how broadly viewers distribute their gaze. This leads to three design relevant implications as follows,

- The first is for situations where a curator or designer wants visitors to follow a more predictable initial reading of a scene. In such cases, preview materials that yield higher baseline correspondence in the early window can be used as candidates for more stable initial orienting. This is a descriptive guideline based on the patterns reported in Sections 3.3 and 3.4.
- The second is for designs that aim to encourage exploration beyond the most visually dominant areas. In that setting, dispersion becomes a useful descriptive pattern. A higher late window gaze entropy indicates broader spatial spread, which may be desirable when the intent is to reduce over concentration on a small number of hotspots and invite scanning across more of the scene.
- The third concerns evaluation before deployment. The present pipeline offers a way to compare candidate pre-viewing materials before deployment, especially when a curator or designer needs to test how different introductory cues shape the first few seconds of scene entry. Section 3.3 can be used to assess whether two preview schemes produce distinct empirical allocation patterns, while Section 3.4 can be used to judge whether differences are most apparent early or whether they emerge later through broader dispersion.

These implications are intended as design-oriented guidance grounded in observed gaze patterns.

### 4.3 Limitations and future research

This study has several limitations. Most importantly, the present block design does not allow briefing modality to be isolated. Specifically, context format is coupled with object category and blocks were presented in a fixed order, so differences observed across blocks cannot be attributed to modality alone. Viewing was task-free and no independent outcome measures were collected (e.g., comprehension or cognitive load), which limits how directly the gaze findings can be linked to interpretive outcomes. The small sample size also limits generalisability and participant-background variables were recorded to characterize the sample, but were not analyzed as moderators. Finally, the stimuli comprised static keyframes extracted from rendered sequences, including consecutive frames, which may introduce dependence between images and familiarity effects (Grill-Spector et al., 2006). The 5 s fixed viewing window was useful for standardizing a short entry phase after preview and for supporting the early-vs.-late analysis, but it does not capture self-paced dwell time, locomotion, repeated returns, or longer interpretive engagement of the kind seen in museum visits. The present findings should therefore be generalized most cautiously to the first moments of orienting after preview, rather than to unrestricted whole-process visit behavior. In immersive (3D-based) viewing, depth-related composition can interact with 2D visual cues and may affect the correspondence between estimated and ground-truth saliency, so domain mismatch may be more pronounced for VR-derived keyframes than for natural-image benchmarks (Celikcan et al., 2020).

DeepGaze III was used as a publicly available pre-trained baseline model originally developed on natural images; any domain mismatch with rendered scenes may affect absolute agreement levels. For this reason, our analysis emphasizes relative changes in gaze-baseline correspondence across blocks rather than absolute model performance. Future work should counterbalance briefing format across object categories, randomize block order, and include at least one behavioral measure (e.g., a brief comprehension or recognition check) to connect gaze patterns to interpretive outcomes.

### 4.4 Conclusion

This study offers an exploratory account of how brief pre-viewing context materials are associated with subsequent attention allocation in rendered museum scenes. Across blocks, the full-window CDS summary suggests the largest overall deviation in the text-only block (Scenario A), an intermediate deviation in the image-only block (Scenario B), and the smallest deviation in the combined text-and-image block (Scenario C). In addition, time-binned profiles indicate that correspondence and dispersion can change over time, with Scenario C showing higher early correspondence but reduced correspondence later, and Scenario B showing the highest late-window gaze dispersion. Nevertheless, combining direct empirical contrasts with time-binned profiling reveals that context-associated differences are not only about baseline fit, but also about when allocation diverges and whether later viewing shifts toward broader dispersion. Future work should use counterbalanced designs and include a simple behavioral check (e.g., recognition) to connect gaze patterns to interpretive outcomes.

## Data Availability

The raw data supporting the conclusions of this article will be made available by the authors, without undue reservation.
